# Relationships between social factors and physical activity among elderly survivors of the Great East Japan earthquake: a cross-sectional study

**DOI:** 10.1186/s12877-016-0203-8

**Published:** 2016-01-27

**Authors:** Eiichi Yoshimura, Kazuko Ishikawa-Takata, Haruka Murakami, Nobuyo Tsuboyama-Kasaoka, Megumi Tsubota-Utsugi, Motohiko Miyachi, Yukari Yokoyama, Kiyomi Sakata, Seiichiro Kobayashi, Akira Ogawa, Nobuo Nishi

**Affiliations:** Faculty of Environmental and Symbiotic Sciences, Prefectural University of Kumamoto, 3-1-100 Tsukide, Higashi-ku, Kumamoto, 862-8502 Japan; Department of Nutritional Education, National Institute of Health and Nutrition, 1-23-1 Toyama, Shinjuku-ku, Tokyo 162-8636 Japan; Department of Health Promotion and Exercise, National Institute of Health and Nutrition, 1-23-1 Toyama, Shinjuku-ku, Tokyo 162-8636 Japan; Department of Nutritional Epidemiology, National Institute of Health and Nutrition, 1-23-1 Toyama, Shinjuku-ku, Tokyo 162-8636 Japan; Center for International Collaboration and Partnership, National Institute of Health and Nutrition, 1-23-1 Toyama, Shinjuku-ku, Tokyo 162-8636 Japan; Faculty of Social Welfare, Nihon Fukushi University, Okuda, Mihama-cho Chita-gun, Aichi, 470-3295 Japan; Department of Hygiene and Preventive Medicine, School of Medicine, Iwate Medical University, 19-1 Uchimaru, Morioka, Iwate 020-8505 Japan; Department of Plastic and Reconstructive Surgery, School of Medicine, Iwate Medical University, 19-1 Uchimaru, Morioka, Iwate 020-8505 Japan; Iwate Medical University, 19-1 Uchimaru, Morioka, Iwate 020-8505 Japan

**Keywords:** Physical activity, Place of residence, Social network, Working status, Disaster, Elderly survivors of the Great East Japan Earthquake

## Abstract

**Background:**

Physical inactivity is a health issue that often occurs after serious disaster. Social factors, which can be disrupted by disaster, are important determinants of physical activity levels in everyday living. This study was designed to confirm the association between social factors and physical activity among elderly survivors of the Great East Japan Earthquake.

**Methods:**

From September 2011 to February 2012, 4316 males and females aged 65 or older participated in a health survey of Great East Japan Earthquake survivors. Multiple logistic regression analyses were performed with the dichotomous dependent variable of physical activity (high versus low) and working status, social network, and place of residence (one’s own home versus elsewhere) as independent variables.

**Results:**

Participants who had been displaced from their homes were more likely to have low physical activity (odds ratio [OR], 95 % confidence interval [CI] for men: 1.37, 1.12 to 1.68; for women: 1.30, 1.09 to 1.55). Non-working status was significantly associated with low physical activity (men: 2.03, 1.65 to 2.49; women: 1.94, 1.60 to 2.34). Detriments to the social network were significantly associated with low physical activity (men: 1.71, 1.41 to 2.08; women: 1.79, 1.51 to 2.13).

**Conclusion:**

Place of residence and social factors were associated with physical activity levels in elderly survivors of the Great East Japan Earthquake. The findings suggest a need for improvement of social factors to encourage increases in physical activity for elderly persons after disaster.

## Background

The Great East Japan Earthquake and Tsunami (GEJET) of 2011 caused damage in Japan (particularly Miyagi, Iwate, and Fukushima Prefecture) on a scale unprecedented for a natural disaster, the effects of which still affect the survivors. As of March 2015, more than 200,000 people have lived in temporary shelters (e.g., temporary housing or relatives’ or acquaintances’ homes).

Our previous study [1] reported that physical activity levels of victims who are living in temporary housing are low compared with national and regional averages. Physical inactivity poses an increased risk for non-communicable disease such as cardiovascular disease, type 2 diabetes, and breast and colon cancers [2]. Furthermore, Ikeda and colleagues [3] have suggested that physical inactivity contributes substantially to mortality from non-communicable disease in Japan. Physical inactivity is the fourth leading risk factor for global mortality, accounting for 6 % of deaths [4]. Increased physical activity is important in terms of public health generally, but physical inactivity is one of the most crucial issues for disaster survivors specifically.

Additionally, physical activity is associated not only with individual factors but also with built environments, and previous studies have indicated that certain factors of the built environment are correlated with physical activity. In a meta-analysis of 16 studies, Duncan et al. [5] reported four such correlates: physical activity facilities, sidewalks, shops and services, and traffic safety. However, current evaluation of perceived environmental factors is difficult because the GEJET disaster area has changed dramatically. In many residential areas, transportation is difficult without a car. Although living in a high-walkability area is conducive to increased walking [6], temporary housing is often set up in neighborhoods with low walkability.

Furthermore, previous studies have indicated a positive relationship between physical activity and social ties [7, 8]. In community-dwelling older adults, the social network has been correlated with facilitation of a physically active lifestyle [9, 10]. It is worth noting that many older adults in the GEJET disaster area lost social supporters, and this reduced social network might be associated with lower physical activity in survivors. Residents of the disaster area who were living in places other than their own homes might experience yet another reduction in social connection. However, in the specific environment of a disaster area, it is presently unclear whether social factors are related to physical activity among survivors. A significant relationship between physical activity and social factors would point up the need for support and encouragement among elderly GEJET survivors.

The objective of the present study was to confirm whether physical activity correlated with social factors such as place of residence, working status, and social network among elderly GEJET survivors.

## Methods

### Study populations

We analyzed part of the data from the RIAS (Research project for prospective investigation of health problems among survivors of the Great East Japan Earthquake and Tsunami Disaster), and the study design has been described previously [11, 12]. The survey was conducted as part of health checkups and employed a common questionnaire inquiring about health conditions and lifestyles. Data collection was carried out between September 2011 and February 2012 in four municipalities in Iwate Prefecture, located in the Tohoku area in the northern part of Honshu, Japan’s largest island. The municipalities were Yamada Town, Otsuchi Town, Kamaishi City, and Rikuzentakata City. These municipalities were heavily damaged by the GEJET. We sent out notifications of the health survey and questionnaires to all residents aged 18 years or older in three of the municipalities. Kamaishi City sent out notifications to residents of temporary housing in the Heita area. A total of 11,103 people underwent health check-ups, and 10,483 people participated in the Health Survey of Great East Japan Earthquake survivors (hereafter, Health Survey). The participation rate was 94.4 %, or about 25 % of the population of that age in the selected area. Of the 10,483 people, we excluded the data of 5,585 people who were 64 years or younger and the data of 582 people who had missing data. Consequently, 4,316 men and women aged 65 or older were analyzed in this study (1,862 men and 2,454 women). The Health Survey was conducted in Yamada Town in September 2011, in Otsuchi Town in December 2011, in Kamaishi City in October 2011, and in Rikuzentakata City between September and October 2011 and in February 2012. The participants gave written informed consent for participation in this study. The study was approved by the ethical committees of both the National Institute of Health and Nutrition and Iwate Medical University.

### Body composition

Height and body weight were assessed during the health checkups, and body mass index (BMI) was calculated as body weight (kg) divided by the square of height (m^2^). Participants’ BMI values were classified as normal (18.5–24.9 kg/m^2^), slim (less than 18.5 kg/m^2^), or overweight/obese (25 kg/m^2^ or more).

### Physical activity

Physical activity was assessed using three questions from the Health Survey. To assess daily physical activity, we asked respondents, “How often do you perform housework and occupational physical activities?” To assess frequency of outings, we asked respondents, ”How often do you go out?” The five response options were: almost every day, three days a week, one day a week, one day a month, and almost never. To assess daily walking time, we asked, “How long do you walk for each day?” The three response options were: more than one hour, thirty minutes to one hour, and less than thirty minutes. Total scores on these questions ranged from 3 to 15 points. Cross-validation of physical activity levels using these items was performed with a triaxial accelerometer (Actimarker EW4800, Panasonic Corporation Japan) [13, 14]. The scores on these questions have been positively correlated with step count (*r* = 0.486) and with amount of moderate-to-vigorous physical activity (*r* = 0.342) [15]. Scores were converted to dichotomous variables based on whether they were above (high physical activity) versus at or below (low physical activity) the median value.

### Social network and other variables

Social network was assessed using Lubben’s social network scale [16, 17], which comprises six Likert-type items with a response scale from 0 to 5. The total score could therefore range from 0 to 30, with higher scores indicating a larger social network size. Lack of social network was classified as a score < 12. Current residence was assessed with six items: own home, home of relative or acquaintance, temporary housing, evacuation center, rented accommodation or new home, or other residence. Place of residence was transformed to a dichotomous variable (own home versus elsewhere) for the present study. To assess standard of living, we asked respondents, “How do you feel about your current economic situation?” The four response options were: severely difficult, difficult, slightly difficult, and acceptable. History of diabetes, hypertension, hyperlipidemia, and depression were assessed as self-reported items (presence or absence). Working status was assessed to measure the socioeconomic factor (working or non-working).

### Statistical analysis

Multiple logistic regression analyses were performed by sex. The dichotomous dependent variable was physical activity (high or low). The independent variables were age in years (65–74, 75–84, ≥ 85), history of disease (prevalence versus absence), standard of living (acceptable, slightly difficult, difficult or severely difficult), place of residence (own home versus elsewhere), BMI (normal, slim, or overweight/obese), working status (working versus non-working) and social network (<12 versus ≥12 ). All analyses were conducted using IBM SPSS Statistics version 23.0 (Tokyo, Japan). A *p* value less than 0.05 was the criterion for statistical significance.

## Results

Table 1 shows the participant characteristics. Men accounted for 43.1 % of the sample. The mean age (SD) was 72.9 (5.4) years. The city of residence was Rikuzentakata for 50.9 %, Yamada for 25.8 %, Otsuchi for 20.7 %, and Kamaishi for 2.6 %. The median physical activity score was 13 for men and 12 for women. The proportion of low physical activity was 47.7 % for men and 54.7 % for women. About 20 % of respondents reported a difficult or severely difficult standard of living. About 40 % of participants lacked a social network (score <12). The fraction of participants who lived in places other than their homes was 37.9 %. The breakdown was 31.2 % in temporary housing, 2.3 % in the home of a relative or acquaintance, 0.4 % in an evacuation center, 3.2 % in a new house or rented accommodation, and 0.8 % in some other type of residence.

**Table 1:** Characteristics of participants

	Men (*n* = 1862)		Women (*n* = 2454)	
	No. of participants	%	No. of participants	%
Physical activity				
High	974	52.3	1111	45.3
Low	888	47.7	1343	54.7
Age group				
65-74 years	1190	63.9	1613	65.7
75-84 years	608	32.7	765	31.2
≥ 85 years	64	3.4	76	3.1
History of disease				
Diabetes	238	12.8	165	6.7
Hypertension	880	47.3	1228	50.0
Hyperlipidemia	114	6.1	374	15.2
Depresion	9	0.5	31	1.3
Body mass index				
Normal body weight	1158	62.2	1539	62.7
Slim	50	2.7	103	4.2
Overweight/Obese	654	35.1	812	33.1
Standard of living				
Acceptable	1040	55.9	1405	57.3
Slightly difficult	483	25.9	645	26.3
Difficult/Severely difficult	339	18.2	404	16.5
Working status				
Working	612	32.9	636	25.9
Non-working	1250	67.1	1818	74.1
Social network				
≥ 12	1126	60.5	1562	63.7
< 12	736	39.5	892	36.3
Place of residence				
Own home	1176	63.2	1503	61.2
Elsewhere	686	36.8	951	38.8

Table 2 shows the odds ratios from multiple logistic regression analysis for low physical activity by individual and social factors for men and women. Increasing age was related to low physical activity levels in both men and women. BMI and standard of living were not associated with physical activity levels. Prevalence of hypertension increased the odds ratio for low physical activity (OR, CI: for men 1.26, 1.04 to 1.53). Non-working status was associated with significantly higher odds ratios for low physical activity among men and women (men: 2.03, 1.65 to 2.49; women: 1.94, 1.60 to 2.34). Detriments in social network were significantly associated with low physical activity among men and women (men 1.71, 1.41 to 2.08; women 1.79, 1.51 to 2.13). Participants who lived in places other than their own homes were also more likely to have low levels of physical activity (men: 1.37, 1.12 to 1.68; women: 1.30, 1.09 to 1.55).

**Table 2:** Odds ratios (95 % CI) for low physical activity by individual and social factors

	Men (*n* = 1862)		Women (*n* = 2454)	
	OR	95 % Cl	OR	95 % Cl
Age group				
65-74 years	1.00		1.00	
75-84 years	1.45	[1.19-1.78]	1.64	[1.36-1.97]
≥ 85 years	2.04	[1.19-3.50]	1.98	[1.18-3.33]
Body mass index				
Normal	1.00		1.00	
Slim	1.08	[0.60-1.96]	0.91	[0.60-1.37]
Overweight/Obese	1.05	[0.86-1.29]	1.10	[0.92-1.32]
History of disease				
Absence of disease	1.00		1.00	
Prevarence of diabetes	1.16	[0.87-1.54]	1.32	[0.94-1.86]
Prevarence of hypertension	1.26	[1.04-1.53]	1.12	[0.95-1.33]
Prevarence of hyperlipidemia	1.21	[0.811.80]	0.95	[0.751.20]
Prevarence of depression	1.51	[0.35-6.44]	2.02	[0.91-4.46]
Standard of living				
Acceptable	1.00		1.00	
Slightly difficult	1.25	[0.99-1.57]	1.18	[0.97-1.44]
Difficult/Severely difficult	1.16	[0.89-1.50]	0.92	[0.73-1.17]
Working status				
Working	1.00		1.00	
Non-working	2.03	[1.65-2.49]	1.94	[1.60-2.34]
Social network				
≥ 12	1.00		1.00	
< 12	1.71	[1.41-2.08]	1.79	[1.51-2.13]
Place of residence				
Own home	1.00		1.00	
Elsewhere	1.37	[1.12-1.68]	1.30	[1.09-1.55]

Abbreviation: *OR* Odds ratio. OR were adjusted for the effect of all other variables shown in table

Table 3 shows the odds ratios from multiple logistic regression analysis for low physical activity by individual and social factors for men and women according to the place of residence. Increasing age was related low physical activity levels. In “own home” group, non-working persons (both men and women) had increased odds ratios for low physical activity (OR, CI for men: 2.09, 1.63 to 2.69; for women: 2.20, 1.75 to 2.76). In “elsewhere” group, non-working status increased the odds ratios of low physical activity of men (OR, CI: 1.96, 1.36 to 2.83). Lack of social network increased the odds ratio for both men and women, regardless of the place of residence (OR, CI for own home group [men: 1.56, 1.23 to 2.00; women: 2.14, 1.71 to 2.68]; for elsewhere group [men: 2.01, 1.46 to 2.79; women: 1.34, 1.02 to 1.77]).

**Table 3:** Odds ratios (95 % CI) for low physical activity by individual and social factors according to place of residence

	Own home				Elsewhere			
	Men (*n* = 1176)		Women (*n* = 1503)		Men (*n* = 686)		Women (*n* = 951)	
	OR	95 % Cl	OR	95 % Cl	OR	95 % Cl	OR	95 % Cl
Age group								
65-74 years	1.00		1.00		1.00		1.00	
75-84 years	1.49	[1.15 - 1.92]	1.55	[1.23 - 1.96]	1.43	[1.02 - 2.02]	1.85	[1.37 - 2.50]
≥ 85 years	2.13	[1.08 - 4.21]	2.21	[1.12 - 4.39]	1.98	[0.81 - 4.84]	1.98	[0.90 - 4.35]
Body mass index								
Normal	1.00		1.00		1.00		1.00	
Slim	0.91	[0.42 - 1.96]	1.02	[0.57 - 1.80]	1.40	[0.53 - 3.70]	0.76	[0.42 - 1.39]
Overweight/Obese	1.05	[0.81 - 1.35]	1.15	[0.92 - 1.44]	1.07	[0.77 - 1.50]	1.03	[0.76 - 1.39]
History of disease								
Absence of disease	1.00		1.00		1.00		1.00	
Prevarence of diabetes	1.38	[0.95 - 1.99]	1.33	[0.86 - 2.08]	1.33	[0.96 - 1.83]	1.27	[0.96 - 1.68]
Prevarence of hypertension	1.23	[0.96 - 1.56]	1.04	[0.84 - 1.30]	0.89	[0.57 - 1.39]	1.29	[0.76 - 2.20]
Prevarence of hyperlipidemia	1.14	[0.71 - 1.84]	1.07	[0.79 - 1.45]	1.33	[0.65 - 2.70]	0.79	[0.55 - 1.14]
Prevarence of depression	1.07	[0.22 - 5.19]	2.76	[0.89 - 8.62]	-#		1.27	[0.41 - 3.98]
Standard of living								
Acceptable	1.00		1.00		1.00		1.00	
Slightly difficult	1.43	[1.06 - 1.92]	1.19	[0.92 - 1.54]	1.05	[0.73 - 1.51]	1.21	[0.88 - 1.65]
Difficult/Severely difficult	1.10	[0.77 - 1.56]	0.85	[0.61 - 1.18]	1.18	[0.79 - 1.74]	1.00	[0.71 - 1.41]
Working status								
Working	1.00		1.00		1.00		1.00	
Non-working	2.09	[1.63 - 2.69]	2.20	[1.75 - 2.76]	1.96	[1.36 - 2.83]	1.39	[0.97 - 1.99]
Social network								
≥ 12	1.00		1.00		1.00		1.00	
< 12	1.56	[1.23 - 2.00]	2.14	[1.71 - 2.68]	2.01	[1.46 - 2.79]	1.34	[1.02 - 1.77]

Abbreviation: OR, Odds ratio. OR were adjusted for the effect of all other variables shown in table

Sharp (#) shows that prevalence of depression was not the analysis (*n* = 2)

## Discussion

The present study examined relationships between physical activity and social factors such as place of residence, standard of living, working status, and social network in the specific environment of a disaster area. One of the main results was that residing somewhere other than their own homes was associated with lower physical activity levels among elderly survivors of the GEJET disaster. This result suggests a particular need to promote physical activity among these elderly survivors. The links between environmental factors and physical activity have been highlighted in recent studies. Residential density, accessibility of destinations, street connectivity, and mixed land use have significant associations with physical activity [18–21]. Recently, Yabuki et al. [22] revealed that survivors living in temporatory housing in Fukushima Prefecture after the GEJET had decreased QOL and lower levels of physical activity compared with national standard values. The GEJET caused critical damage in three prefectures (Iwate, Miyagi, and Fukushima). Fukushima Prefecture has a serious double burden of a nuclear power plant accident and the tsunami. Therefore, social situations and physical activity might be different for survivors in this prefecture. Future study is needed to clarify the influences on social situation and physical activity in these prefectures, compared with non-disaster areas. However, to our knowledge, no study has focused on physical activity and place of residence in elderly survivors. The present study was not able to assess activity type and environmental factors objectively, although daily physical activity appeared to be lower for survivors who lived in places other than their own homes. Because many residences in the disaster area are low-walkability neighborhoods, this is an important issue for public health, and an approach for encouraging increased physical activity in those survivors would be desirable. There is also a need for future studies to clarify the contributions of individual and environmental factors to physical activity.

Previous studies have indicated that lack of social network is associated with low physical activity and function [7, 8, 23], and our results are consistent with this finding. If elderly persons desire to increase their physical activity, they require social support from others close to them [24]. Intervention measures and policies will be needed for creating good social networks (e.g., connections among individuals) so that physical activity and function can be increased. We had assumed that social network would be associated with place of residence; however, chi-square tests revealed no significant link (men: *p* = 0.768, women: *p* = 0.058). Otherwise, the relationship between physical activity and social network was significant, regardless of the place of residence. Therefore, in the specific environment of a disaster area, social network and place of residence might be independent predictors of physical activity. However, further research is necessary to consider the relationships among physical activity, social network, and place of residence. Non-working status has previously been associated with low physical activity [25]. The present study also showed that non-working persons were more likely to have low physical activity levels. Improvement of socioeconomic status, including working status, would be important for increased physical activity.

The present study has some limitations. First, participants’ physical activity levels were converted to dichotomous variables based their relationship to the median value (high versus low). Therefore, physical activity levels were not quantified to the extent that would be attained with instruments such as an accelerometer (e.g., metabolic equivalents per hour). However, the questionnaire we used has been validated as producing significant correlations with both step count and amount of moderate-to-vigorous physical activity [15]. Second, this study assessed place of residence as the only environmental characteristic. Further research should assess the neighborhood environment objectively with the help of geographic information systems. Third, causal relationships cannot be inferred from the results of this study because of its cross-sectional nature. Fourth, although the participants in this study were elderly survivors of the GEJET, the relationship between various social factors and physical activity may be different in a disaster area compared with a non-disaster area. A challenge for future study is to design a comparison of disaster and non-disaster areas. Nevertheless, this study was a meaningful beginning in the exploration of individual and environmental factors that affect physical activity among elderly survivors of a natural disaster.

## Conclusion

The present study indicated that being displaced from one’s residence has an effect on physical activity among elderly GEJET survivors. We also showed that lack of social connections is associated with low physical activity levels in this population. The results may suggest a need to provide social supports for increasing physical activity among elderly survivors of natural disaster.
